# Melanoma risk, tumour stage, and melanoma-specific mortality in individuals with diabetes: a systematic review and meta-analysis

**DOI:** 10.1186/s12885-024-12598-8

**Published:** 2024-07-07

**Authors:** Jens Ejrnæs Tønder, Marie Louise Bønnelykke-Behrndtz, Tinne Laurberg, Eeva-Liisa Røssell, Martin Sollie

**Affiliations:** 1 Department of Plastic and Breast Surgery, Aarhus University Hospital, Palle Juul-Jensens Boulevard 99, Aarhus N, 8200 Denmark; 2 Steno Diabetes Center Aarhus, Aarhus University Hospital, Aarhus, Denmark; 3 Department of Pathology, Aarhus University Hospital, Aarhus, Denmark; 4 Department of Public Health, Aarhus University, Aarhus, Denmark

**Keywords:** Melanoma, Diabetes, Type 2 diabetes, Cancer, Systematic review, Meta-analysis

## Abstract

**Background:**

Cancer has become the leading diabetes-related cause of death in high-income countries, and more knowledge is needed to clarify the impact of diabetes on site-specific cancers. The purpose of this study is to assess the association between diabetes and malignant melanoma by conducting a comprehensive systematic review and meta-analysis.

**Methods:**

Using predefined eligibility criteria, PubMed, The Cochrane Library and Web of Science were systematically searched up to February 22, 2023. Exposure was defined as diabetes or type 2 diabetes and the outcomes were defined as melanoma incidence, melanoma stage or melanoma-specific mortality. The identified articles were evaluated by two independent reviewers and quality assessment was conducted using the Newcastle-Ottawa Scale for observational studies. Meta-analyses were conducted using RevMan 5.4.1 on melanoma risk using adjusted risk estimates and on melanoma stage using a dichotomous model.

**Results:**

The literature search revealed 20 studies in total eligible for inclusion, 14 for the analysis of melanoma risk, 3 for melanoma thickness and ulceration, and 4 for melanoma-specific survival. According to the meta-analyses, diabetes did not impact the risk of developing melanoma (RR:1.05, 95%CI:0.99–1.12, *p* = 0.10). However, type 2 diabetes was associated with more advanced melanoma stages at the time of diagnosis (Breslow-thickness > 1 mm: RR 1.35, 95%CI: 1.22–1.49, *p* = < 0.001) and presence of ulceration (RR 1.30, 95%CI: 1.00-1.68, *p* = 0.05). A meta-analysis on the association between diabetes and melanoma-specific mortality was not feasible due to diverse study designs.

**Conclusion:**

Our meta-analysis found no association between diabetes and the risk of developing melanoma, but diabetes was associated with increased tumour thickness and the presence of ulceration at the time of diagnosis. Further research is warranted to explore the association between diabetes melanoma stage and prognosis.

**Trial registration:**

PROSPERO ID CRD42023394187.

**Supplementary Information:**

The online version contains supplementary material available at 10.1186/s12885-024-12598-8.

## Introduction

Melanoma is the sixth most frequent type of cancer in Europe, and incidence has rapidly increased over the last few decades [1, 2]. Melanoma is a multi-factorial disease with a combined genetic and environmental aetiology [3–5]. The primary risk factor, widely acknowledged, is intermittent exposure to ultraviolet (UV) radiation and a history of sunburns across all age groups [4, 5]. Increased risk is also observed in light-skinned individuals [6] and individuals with a family history of melanoma [3].

Type 2 diabetes mellitus (T2DM) is a disease with rapidly increasing incidence worldwide [7]. The number of cases has doubled from 11.3 million in 1990 to 22.9 million in 2017, and this upward trend is expected to continue [7]. Since the beginning of the millennium, the leading contributor to diabetes-related death in high-income countries has changed from cardiovascular disease to cancer [8]. Several molecular mechanisms have been proposed to explain the carcinogenic effect of T2DM, including chronic systemic inflammation, hyperinsulinemia, and hyperglycaemia [9]. These same mechanisms have also been linked to increased melanoma aggressiveness in mice with diabetes [10].

With the increased incidence of T2DM and melanoma, a rise in the number of individuals diagnosed with both conditions is expected [2, 7]. Prior studies have suggested that diabetes may be associated with an increased risk of melanoma [11], and in breast cancer studies, diabetes has been shown to predispose to a more aggressive cancer type [12, 13]. However, research on the association between diabetes and melanoma has been limited by the lack of extensive register-based studies. To provide robust risk estimates with large study populations, we conducted a systematic review and meta-analysis to quantify the association between diabetes and the risk of melanoma, melanoma stage, and melanoma-specific mortality, respectively.

## Methods

### Data sources

We conducted our review based on the Preferred Reporting Items for Systematic Reviews and Meta-Analyses (PRISMA) guidelines [14] and followed a publicly available protocol to guide the systematic review [15] (PROSPERO: CRD42023394187). The literature search was performed in PubMed, The Cochrane Library, and Web of Science with the search string: “((Diabetes Mellitus [MeSH Terms]) OR (Diabetes)) AND ((Malignant Melanoma [MeSH Terms]) OR (Melanoma) OR (Malignant Melanoma) OR (Cutaneous Melanoma))” up to the 22nd of February 2023. The search was limited to full texts in English and included randomised controlled trials, cohort studies, case-control studies, and reviews. To supplement our search and ensure saturation, reference lists of the included studies and literature reviews were manually searched.

### Study selection and eligibility criteria

Two independent reviewers (JT and MS) assessed the identified papers using the Covidence software. The reference lists of included publications were manually screened, and relevant titles were evaluated for potential inclusion. Study eligibility criteria were as follows: (1) the exposure must be defined as either diabetes or type 2 diabetes; (2) the comparison group must be non-diabetics; and (3) outcomes are defined as melanoma incidence, melanoma stage, or melanoma-specific mortality. Studies exclusively focusing on type 1 diabetes were excluded due to the difference in pathophysiology and age of onset between type 1 and type 2 diabetes [16].

### Quality assessment

All included studies were assessed for quality using the Newcastle-Ottawa Scale [17], designed for non-randomised study evaluation in meta-analyses. The scale utilises a point-based system ranging from 0 to 9 points to assess the quality of studies based on three broad perspectives: selection of participants, comparability of groups, and ascertainment of exposure and outcome. Cross-sectional studies were assessed with an adapted version of the Newcastle-Ottawa scale [18], ranging from 0 to 10 points. The studies needed a minimum score of seven points to be eligible for inclusion in the meta-analyses.

### Data extraction

Data were extracted using a predefined spreadsheet and included the author’s name, publication year, country of study, study type, study population, population size, study period, methods for ascertainment of diabetes diagnosis, classification of diabetes type, age, sex, length of the follow-up period, adjustments made for relevant factors, melanoma tumour thickness, presence of ulceration, melanoma risk, and melanoma-specific mortality.

### Statistical analyses

Statistical analyses were conducted using RevMan software version 5.4.1 [19]. We used risk ratios (RRs) to examine melanoma risk in our analyses. If the included studies reported standardised incidence rates (SIRs) or incidence rate ratios (IRRs) we considered them directly as RRs. The analysis was performed using the inverse variance statistical method and applying a random effects analysis model to account for heterogeneity according to the DerSimonian and Laird method [20, 21]. Subgroup analyses were performed by sex, diabetes specification, study quality and follow-up time.

To analyse the association between diabetes and Breslow tumour thickness and ulceration, we constructed a database incorporating data from the relevant studies. Two dichotomous models were constructed to analyse the risk of tumour thickness exceeding 1 mm and the risk of having ulcerated melanoma at the time of diagnosis. For these analyses, we calculated RRs using the Mantel-Haenszel method and applied a random effects analysis model.

## Results

### Included studies and data

**Fig. 1:**
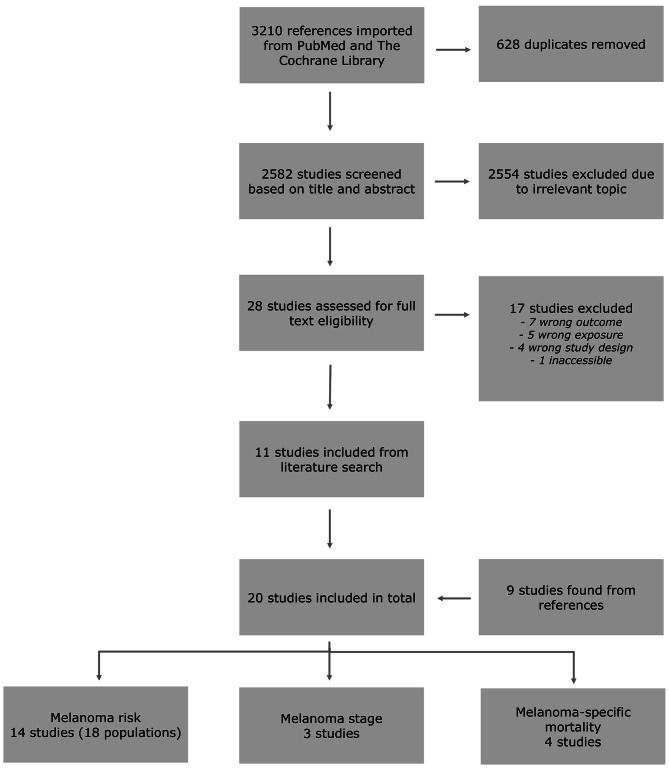
Flowchart of study screening and assessment for inclusion in the study. One study was eligible in both the analysis of melanoma risk and melanoma-specific mortality

The literature search revealed 2582 unique records, of which eleven were included in the study. The assessment of full text revealed nine studies in references eligible for inclusion in the review, concluding the literature search with a total of twenty studies included (Fig. 1).

Individual study characteristics are presented in Tables 1, 2 and 3. Thirteen studies examined melanoma risk [22–34], three examined melanoma stage [35–37], three examined melanoma-specific mortality alone [38–40], and one study examined both melanoma risk and melanoma-specific mortality [41].

**Table 1:** Study characteristics of studies included in the analysis of melanoma risk:

Year	Author	Country	Type of study	Study population	Study period	Population size	Diabetes type	Diabetes diagnosis	Age in years	Gender	Follow-up	Adjustment
2019	Linkeviciute-Ulinskiene, D [22].	Lithuania	Retrospective cohort study	Individuals with diabetes	2000–2012	48,467	T2DM	Register	Mean: 59.7	Men	12 years	Age, sex
2019	Linkeviciute-Ulinskiene, D [22].	Lithuania	Retrospective cohort study	Individuals with diabetes	2000–2012	78,823	T2DM	Register	Mean: 64	Women	12 years	Age, sex
2019	Saarela, K [34].	Finland	Retrospective cohort study	Individuals with diabetes	1989–2014	428,326	T2DM	Register	> 30	Both	Mean: 10.5 years	Age in 5-year brackets, sex
2016	Gini, A [29].	Italy	Retrospective cohort study	Individuals with diabetes	2002–2009	32,247	T2DM	Register	40–85	Both	Median: 3.65 years	Age, sex, year of cancer diagnosis
2016	Tseng, HW [26].	China (Taiwan)	Retrospective cohort study	Individuals with diabetes	2000–2012	41,898	Undefined	Register	Mean: 57.4	Both	N/a	Age, sex, index date, and comorbidities
2015	Harding, JL [41].	Australia	Retrospective cohort study	Individuals with diabetes	1997–2008	872,706	T2DM	Register	Mean: 59.5	Both	Median: 5.8 years	Calendar year, age in 5-year brackets, sex
2015	Liu, X [30].	Sweden	Retrospective cohort study	Individuals with diabetes	1964–2010	380,196	T2DM	Register	Median: 69	Both	Median: 4 years	Age in 5-year brackets, sex, year in 5-year brackets, region, socioeconomic status
2013	Walker, J.J [23].	Scotland	Retrospective cohort study	Individuals with diabetes	2001–2007	44,246	T2DM	Register	55–79	Men	N/a	Age, sex
2013	Walker, J.J [23].	Scotland	Retrospective cohort study	Individuals with diabetes	2001–2007	36,592	T2DM	Register	55–79	Women	N/a	Age, sex
2012	Attner, B [31].	Sweden (Scania)	Case-control study	Individuals with diabetes	1998–2007	17,032	Undefined	Register	45–84	Both	4–10 years	Age, sex and domicile
2012	Lo, S [32].	China (Taiwan)	Retrospective cohort study	Individuals with diabetes	1996–2009	895,434	T2DM	Register	Mean: 60.5	Both	Median: 3.5 years	Age, sex, year of diabetes diagnosis
2011	Atchison, EA [33].	U.S.	Retrospective cohort study	U.S. Veterans admitted to Veterans Affairs hospitals.	1969–1996	594,815	Undefined	Register	Mean: 52.3	Men	1–30 years	Age, time, latency, race, number of visits, diagnoses of alcohol–related conditions, obesity, and chronic obstructive pulmonary disease
2011	Wotton, CJ [24].	England	Retrospective cohort study	Individuals with diabetes	1963–1998	15,898	Undefined	Register	≥ 30	Both	1–35 years	Age in 5-year brackets
2011	Wotton, CJ [24].	England	Retrospective cohort study	Individuals with diabetes	1999–2008	7,771	Undefined	Register	≥ 30	Both	1–9 years	Age in 5-year brackets
2010	Hemminki, K [27].	Sweden	Retrospective cohort study	Individuals with diabetes	1964–2007	125,126	T2DM	Register	> 39	Both	Median: 15 years	Age, sex, period, region, socioeconomic status, obesity
2009	Yood, MU [28].	Sweden	Retrospective cohort study	Individuals with diabetes	2000–2004	191,223	T2DM	Register	Mean DM: 56.2, non DM: 53.1	Both	Mean: 3.5 years	Age, sex, and selected cancer risk factors.
1997	Wideroff, L [25].	Denmark	Retrospective cohort study	Individuals with diabetes	1977–1993	54,571	Undefined	Register	Median: 64	Men	Mean: 5.7 years	Sex
1997	Wideroff, L [25].	Denmark	Retrospective cohort study	Individuals with diabetes	1977–1993	55,010	Undefined	Register	Median: 69	Women	Mean: 5.7	Sex

**Table 2:** Study characteristics of studies included in the analysis of melanoma stage:

Year	Author	Country	Type of study	Study population	Study period	Population size	Diabetes type	Diabetes diagnosis	Age in years	Gender	Follow-up	Adjustment
2022	Spoerl, S [36].	Germany (Eastern Bavaria)	Retrospective cohort study	Patients with head- and neck melanoma	2010–2017	382	T2DM	Medical records	Mean 69.2	Both	Mean 4.5 years	Age, BMI, statin use, intake of ASA, tumour size, nodal status, ulceration, and distinct tumour histologic types
2022	Straker RJ [37].	USA	Retrospective cohort study	Patients with melanoma stage 1 or 2 undergoing sentinel node biopsy	2007–2016	1128	T2DM	Medical records	Mean 58.9	Both	Median 7 years	Age, insulin use, sex, race, tumour location, histology, Breslow thickness, ulceration, mitoses, lymphocyte infiltration, lymphovascular invasion, microsatellites, sentinel lymph node status, lymph node dissection, adjuvant systemic therapy
2021	Nagore, E [35].	Spain	Retrospective cross-sectional study	Patients with melanoma	2012–2015	443	T2DM	Blood sample and former diagnosis or medication	Mean 55.98	Both	N/a	Sex, BMI, alcohol, smoking

**Table 3:** Study characteristics of studies included in the analysis of melanoma specific mortality:

Year	Author	Country	Type of study	Study population	Study period	Population size	Diabetes type	Diabetes diagnosis	Age in years	Gender	Follow-up	Adjustment
2012	Liu, X [39].	Sweden	Retrospective cohort study	Patients with all-type cancer	1961–2008	1,016,105	T2DM	Register	N/a	Both	Mean 7 years	Age at diagnosis, sex, period, obesity, alcohol, smoking, socioeconomic status, and diagnosis region.
2020	Urbonas, V [40].	Lithuania	Retrospective cohort study	Patients with melanoma	2001–2013	3530	T2DM	Register	Mean: Diabetics: 69 Non-diabetics: 58	Both	> 6 months	Gender, age group, and stage at diagnosis
2004	Coughlin, S [38].	USA	Prospective cohort study	Adult volunteers	1982-	1,056,243	Undefined	Questionnaire	Mean 57	Both	16 years	Age
2015	Harding, JL [41].	Australia	Retrospective cohort study	Individuals with diabetes	1997–2008	872,706	T2DM	Register	Mean: 59.5	Both	Median: 5.8 years	Calendar year, age in 5-year brackets, sex

Eighteen studies were cohort studies [22–30, 32–34, 36–40], one was case-control [31], and one was a cross sectional study [35]. Fourteen were conducted in Europe [22–25, 27, 28, 30, 31, 34–37, 39, 40], three in USA [33, 37, 38], two in Asia [26, 32], and one in Australia [41]. Study periods ranged from 1961 to 2017, and the population sizes ranged from 382 to 1,056,243 individuals. All twenty studies met the minimum requirement of seven points on the Newcastle-Ottawa scale (Supplementary 1 and 2).

### Assessment of the exposure

Diabetes status was determined from registry data in sixteen studies [22–34, 39–41]. Eleven studies solely considered International Classification of Diseases (ICD) codes [23–27, 30–33, 39, 41], of which six were from hospital discharge records [24, 25, 27, 30, 33, 39], two from national insurance funds [26, 32], two from national diabetes registries [23, 41], and one from health care registries [31]. Five studies considered prescription records in combination with ICD codes, of which three considered national health insurance funds [22, 28, 40], one considered hospital discharge records [29], and one national diabetes registry [34].

Among the remaining four studies [35–38], two considered medical records [36, 37], one considered fasting plasma glucose from a blood sample combined with medical records [35], and one considered self-reported diabetes status [38]. Fourteen studies specifically defined T2DM as the exposure [22, 23, 27–30, 32, 34–37, 39–41], whereas the remaining six studies did not specify the type of diabetes investigated [24–26, 31, 33, 38].

### Diabetes and melanoma risk

**Fig. 2:**
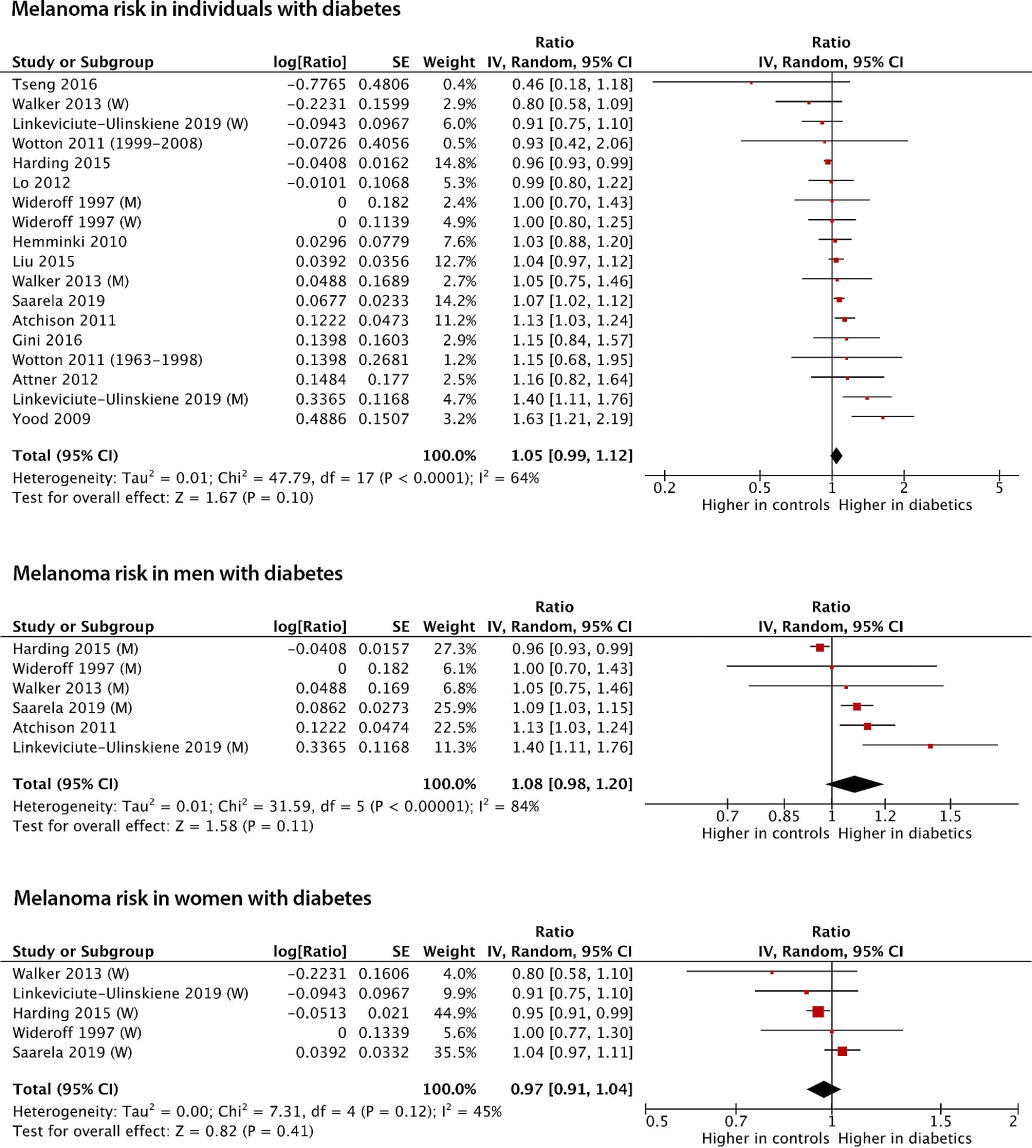
Forest plots of the meta-analyses of the association between diabetes and overall melanoma incidence, melanoma incidence in men, and melanoma incidence in women. Five studies reported stratified risk measurements based on sex [22, 23, 25, 34, 41] and one study cohort only included men [33]. Risk measurements including 95% CIs are reported on a logarithmic scale. IV: inverse variance. (M): Men. (W): Women

Fourteen studies comprising eighteen populations with a total of 3,920,281 individuals were eligible for inclusion in the meta-analysis assessing the risk of melanoma (Table 1). The incidence of melanoma was determined solely from ICD codes in twelve studies [22–25, 27, 29–34, 41], of which ten considered national cancer registries [22, 23, 25, 27, 29–32, 34, 41], one admission records [24], and one discharge records [33]. One study considered ICD codes in a cancer registry combined with in/outpatient visits in national health insurance funds [26], and one ICD codes combined with procedure codes for melanoma excision in national health insurance funds [28]. In four distinct cohorts [22, 28, 33, 34], an increased risk of melanoma was observed among individuals with diabetes. In contrast, a reduced risk was found in one cohort [41], and no association between diabetes and melanoma risk was found in the remaining 13 cohorts [22–32]. The population sizes ranged from 7,771 to 895,434 participants, and the risk estimates ranged from 0.46 to 1.63. The meta-analysis examining melanoma risk showed no significant difference in the risk of developing melanoma when comparing individuals with and without diabetes (RR: 1.05 (95%CI: 0.99–1.12, *p* = 0.10)) (Fig. 2). Five studies provided stratified analyses based on sex [22, 23, 25, 34, 41], and one study cohort consisted entirely of men [33]. In the sex-stratified meta-analyses of melanoma risk, men with diabetes exhibited a RR of 1.08 (95%CI: 0.981.20, *p* = 0.11), while women with diabetes had a RR of 0.97 (95%CI: 0.91–1.04, *p* = 0.41) when compared with men and women without diabetes, respectively (Fig. 2). In the subgroup analyses the results remained consistent (Fig. S1, S2 and S3).

### Type 2 diabetes and melanoma stage

Three studies with a total of 1953 patients with melanoma examined the association of specifically T2DM and melanoma stage [35–37](Table 2). Melanoma stage was determined from histopathological slides in two studies [35, 37] and medical records in one study [36]. Among these studies, two reported an increased risk of having Breslow tumour thickness >1 mm among individuals with T2DM [35, 37], and one found no association of T2DM with tumour thickness [36]. Additionally, one of the three studies found an increased risk of ulceration in individuals with T2DM [37]. The study population ranged from 382 to 1128 participants. RRs of the risk of melanoma thickness > 1 mm in our dichotomous model ranged from 1.12 to 1.44 and from 1.04 to 1.42 for the risk of ulceration in individuals with T2DM compared to those without T2DM.

**Fig. 3:**
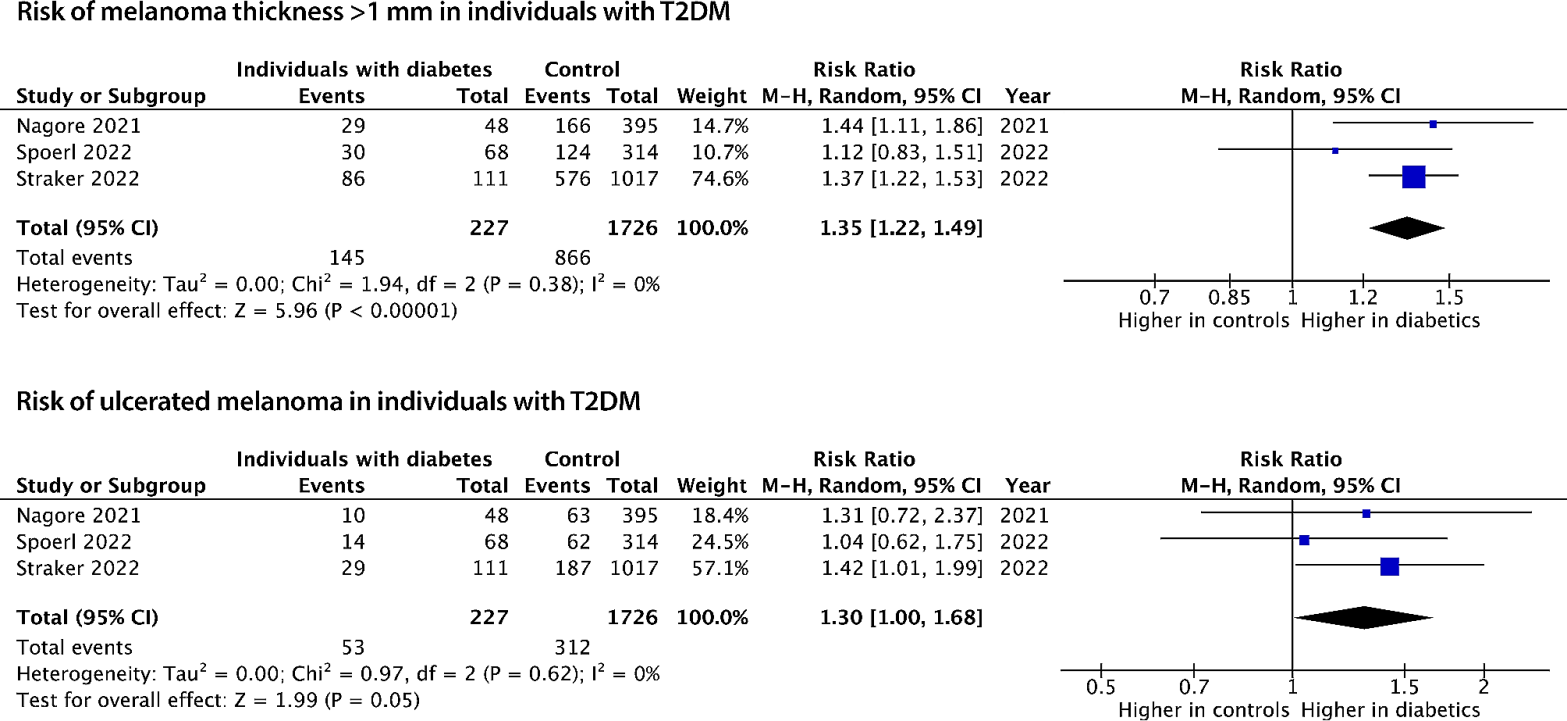
Forest plots of the meta-analyses of the association between T2DM, Breslow tumour thickness and presence of ulceration at the time of diagnosis. Event is defined as tumour thickness > 1 mm and presence of ulceration. M-H: Mantel-Haenszel

The analyses of melanoma stage in individuals with T2DM showed an increased risk of being diagnosed with Breslow tumour thickness > 1 mm (RR: 1.35 (95%CI: 1.22–1.49, *p* = < 0.001)) and presence of ulceration (RR 1.30, 95%CI: 1.00-1.68, *p* = 0.05)) compared with individuals without T2DM (Fig. 3).

### Diabetes and melanoma-specific mortality

Four studies with a total population size of 2,948,584 met the review criteria and were eligible for further analysis of the association between diabetes and melanoma-specific mortality [38–41](Table 3). Melanoma-specific mortality was determined using ICD-codes in all four included studies [38–41], of which three used national cause-of-death indexes [38, 39, 41] and one used a national cancer registry [40]. No meta-analyses of the association between diabetes and melanoma-specific mortality were performed due to the heterogeneity of study designs. First, the study populations were not comparable; two were national register-based cohorts, one was melanoma patients, and one was a health research cohort with a voluntary enrolment of adults older than 35. The reported outcomes diverged in terms of melanoma-specific survival and overall survival, and the risk measurements reported varied between Kaplan-Meier survival plots, standardised mortality rates, RRs, and hazard ratios.

An association between diabetes and the prognosis of patients with melanoma across four studies was not found. Specifically, two studies indicated that T2DM was associated with lower melanoma-specific mortality [40, 41], while the other two studies found no impact of diabetes on cancer-specific mortality [38, 39]. The heterogeneity in study designs might explain the differing results found across studies.

## Discussion

In this systematic review, we examined the association between diabetes and melanoma risk, melanoma stage (Breslow thickness and ulceration), and melanoma-specific mortality. Twenty papers, including more than 5.9 million individuals, were included in the analyses. Our findings suggest that while there is no significant association between diabetes and melanoma risk or melanoma-specific mortality, those with T2D are at a higher risk of being diagnosed with more advanced stages of melanoma.

### Melanoma risk

The impact of diabetes on the risk of developing melanoma was estimated based on eighteen cohorts with a cumulative sample size of 3,920,281 individuals, and no association was found. When we conducted analyses stratified by sex, similar results were found. Our findings on melanoma risk are consistent with a prior, smaller meta-analysis conducted by Ling et al. [42]. In their study, Ling et al. summarised results from eleven studies, nine of which were also included in our analysis. They reported a RR of 1.06 (95% CI: 0.95–1.19). Additionally, Qi et al. observed a modest increase in melanoma risk (RR 1.15, 95% CI: 1.00-1.32) among individuals with diabetes compared to non-diabetic individuals across nine studies, six of which overlapped with our analysis [11]. The association between diabetes and cancer risk have been investigated in several high-quality register studies with large populations showing an increased risk of overall cancer and several site-specific cancers [42]. Despite the heightened overall cancer risk among individuals with diabetes, our meta-analysis of eighteen cohorts found no evidence to support an association between diabetes and the risk of melanoma.

### Melanoma stage

Tumour thickness and ulceration are important prognostic indicators in melanoma [43]. Our meta-analysis examining the association between T2DM and tumour thickness found a 35% increased risk of having tumour thickness > 1 mm among individuals with T2DM. Furthermore, the risk of being diagnosed with an ulcerated melanoma exhibited a similar increased risk, with a 30% increased risk observed in individuals with T2DM compared with those without T2DM.

The increased tumour progression observed in individuals with diabetes can be attributed to several proposed mechanisms. Long-lasting hyperglycaemia can affect cell growth and cause DNA damage [44]. Hyperinsulinemia caused by insulin resistance leads to higher levels of insulin-like growth factor 1, which has been proposed to contribute to a pro-tumoral microenvironment [10, 45]. Chronic inflammation, a hallmark of cancer [46], is associated with T2DM-induced immunosuppression, causing dysfunction of CD8 + T cells [10, 47]. This dysfunction may be associated with tumour growth [10]. In a T2DM and melanoma aggressiveness study in a mouse diabetes model, increased melanoma growth was found in diabetic mice [10].

### Strengths and limitations

The major strength of our meta-analysis on the risk of developing melanoma in individuals with diabetes lies in the size of the total population and the high quality of the included studies. Ascertainment of diabetes status was register-based in all included studies for the melanoma risk analyses, eliminating the risk of bias from self-reported diabetes status. All the included studies adjusted for sex, and seventeen of the included eighteen studies adjusted for age; both proposed risk factors for melanoma [48]. Having mostly register-based data also provides some limitations, as none of the studies adjusted for UV radiation exposure or genetic disposition, which are known risk factors for melanoma [5, 6]. Additionally, none of the studies adjusted for lifestyle factors such as smoking, alcohol consumption, and physical activity, which may impact melanoma development [5, 6, 48]. Although lifestyle factors do not affect the melanoma risk [6], they may be associated with more advanced stages at diagnosis [49]. Thus our findings may be affected by confounding. However, Nagore et al. [35] found that T2D was independently associated with advanced stages of melanoma at the time of diagnosis, even after adjusting for smoking and alcohol use.

The studies based on hospital registers may underestimate the association between diabetes and melanoma risk due to individuals receiving outpatient care for their diabetes in the background population. Additionally, the majority of populations included in these studies are from high-income countries, with only two Asian populations represented and no African or Hispanic populations included. As a result, the generalizability of these findings may be limited. However, the populations of the included studies do represent those with the highest incidence of melanoma, which is typically seen in individuals with light skin of Caucasian origin [6, 48]. Furthermore, in the meta-analysis of melanoma characteristics in individuals with T2DM, although a low heterogeneity was observed among the included studies, only three reported comparable outcomes. Therefore, additional studies of tumour characteristics are necessary to support the findings of this meta-analysis regarding the increased melanoma stage in individuals with T2DM.

The analysis of melanoma-specific mortality was limited by the heterogeneity of the included studies. Additionally, three of the studies that were included were cohort studies investigating the association between diabetes and cancer-specific mortality across various site-specific cancers [38, 39, 41], so an in-depth analysis of the correlation with melanoma-specific mortality was not conducted. Urbonas et al. [40] examined the association between T2D and specifically melanoma-specific mortality. However, their findings were limited by a small number of patients with diabetes (*n* = 163), which limited the extent of their analysis.

### Clinical implications and further research

This study highlights the need for increased awareness of early detection of melanoma among individuals with T2D to ensure they receive the best possible care. Furthermore, it also identifies significant knowledge gaps regarding the association between diabetes and melanoma. Population-based studies with large populations are warranted to support the findings of increased melanoma stages at the time of diagnosis among individuals with T2D. Additionally, extensive cohort studies of patients with melanoma are needed to investigate the impact of T2D on melanoma-specific survival and to assess whether a potential association is stage-specific.

## Conclusion

In conclusion, our meta-analyses of eighteen studies did not find an association between diabetes and the risk of developing melanoma. However, individuals with T2DM were found to have a higher risk of being diagnosed with tumour thickness > 1 mm, and a similar trend was observed for the presence of ulceration when compared with individuals without T2DM. The risk assessment of the association between diabetes and melanoma-specific mortality could not be conducted due to the heterogeneity of study designs. Further studies with large population sizes, high data quality, and long follow-up periods are warranted to increase our understanding of how diabetes impacts patients with melanoma.

### Electronic supplementary material

Below is the link to the electronic supplementary material.

Supplementary Material 1

## Data Availability

The data analysed in this manuscript is gathered from publicly available articles. The datasets analysed in the current study are available from the corresponding author on reasonable request.
