# Supplementary material for: Melanoma risk, tumour stage, and melanoma-specific mortality in individuals with diabetes: a systematic review and meta-analysis
Source: BMC Cancer. 2024 Jul 7;24:812. doi: 10.1186/s12885-024-12598-8 (PMC11229239; doi:10.1186/s12885-024-12598-8)
Supplement: Supplementary file 1 — Supplementary Material 1 [file 12885_2024_12598_MOESM1_ESM.pdf]

Supplementary 1 – Quality assessment of cohort studies:

Study	Selection				Comparability		Outcome			
	**A**	**B**	**C**	**D**	**E**	**F**	**G**	**H**	**I**	**Total**
Hemminki, K.(27)	1	1	1	1	1	1	1	1	1	9
Atchison, EA.(33)	1	1	1	1	1	1	1	1	1	9
Wotton, CJ.(24) (1963-1998)	1	1	1	1	1	0	1	1	0	7
Wotton, CJ.(24) (1999-2008)	1	1	1	1	1	0	1	0	1	7
Harding, JL.(41)	1	1	1	1	1	0	1	1	1	8
Tseng, HW.(26)	1	1	1	1	1	0	1	1	1	8
Linkeviciute-Ulinskiene, D.(22)	1	1	1	1	1	0	1	1	1	8
Saarela, K.(34)	1	1	1	1	1	0	1	1	1	8
Urbonas, V.(40)	1	1	1	1	1	0	1	1	1	8
Yood, MU.(28)	1	1	1	1	1	0	1	0	1	7
Wideroff, L.(25)	1	1	1	1	1	0	1	1	0	7
Liu, X.(39)	1	1	1	1	1	1	1	1	1	9
Coughlin, SS.(38)	1	1	1	1	1	1	1	1	1	9
Gini, A.(29)	1	1	1	1	1	0	1	0	1	7
Walker, J.J.(23)	1	1	1	1	1	0	1	0	1	7
Attner, B.(31)	1	1	1	1	1	0	1	1	1	8
Lo, S.(32)	1	1	1	1	1	0	1	1	1	8
Liu, X.(30)	1	1	1	1	1	0	1	0	1	7

Supplementary 1. Quality assessment of the included observational studies utilizing the Newcastle-Ottawa Scale(17). A: Exposed cohort representative of the average in the community. B: Non-exposed cohort drawn from the same community. C: Ascertainment of exposure from secure record or structured interview. D: Demonstration that outcome was not present at start of study. E: Study controls for age and sex. F: Study controls for lifestyle factors (smoking, alcohol consumption, obesity). G: Assessment of outcome of high quality. H: Follow up >5 years. I: Adequate cohort follow up.

Supplementary 2 – Quality assessment of cross-sectional studies:

Study	Selection				Comparability	Outcome		
	**A**	**B**	**C**	**D**	**E**	**F**	**G**	**Total**
Nagore, E.(35)	1	1	1	2	1	2	1	9
Spoerl, S.(36)	1	1	1	2	1	2	1	9
Straker, RJ.(37)	1	1	1	2	1	2	1	9

Supplementary 2.Quality assessment of included cross sectional studies utilizing the adapted Newcastle-Ottawa scale for cross sectional studies(18). A Sample representative of the target population. B: Sample size justified and satisfactory. C: Comparability between respondents and non-respondents' characteristics. D: Ascertainment of exposure: validated measurement tool = 2 points. E: Study controls for age and sex = 1, study controls for lifestyle factors (smoking, alcohol consumption, obesity) = 1. F: Assessment of outcome independent blinded or record linkage = 2. G: Statistical test clearly described, and p value stated.

Type 2 diabetes and melanoma incidence

Study or Subgroup	log[Risk Ratio]	SE	Weight	Risk Ratio IV, Random, 95% CI
Walker 2013 (W)	−0.2231	0.1599	3.9%	0.80 [0.58, 1.09]
Linkeviciute–Ulinskiene 2019 (W)	−0.0943	0.0967	8.0%	0.91 [0.75, 1.10]
Harding 2015	−0.0408	0.0162	18.7%	0.96 [0.93, 0.99]
Lo 2012	−0.0101	0.1068	7.1%	0.99 [0.80, 1.22]
Hemminki 2010	0.0296	0.0779	10.0%	1.03 [0.88, 1.20]
Liu 2015	0.0392	0.0356	16.3%	1.04 [0.97, 1.12]
Walker 2013 (M)	0.0488	0.1689	3.6%	1.05 [0.75, 1.46]
Saarela 2019	0.0677	0.0233	17.9%	1.07 [1.02, 1.12]
Gini 2016	0.1398	0.1603	3.9%	1.15 [0.84, 1.57]
Linkeviciute–Ulinskiene 2019 (M)	0.3365	0.1168	6.3%	1.40 [1.11, 1.76]
Yood 2009	0.4886	0.1507	4.3%	1.63 [1.21, 2.19]
**Total (95% CI)**			**100.0%**	**1.05 [0.98, 1.12]**

Heterogeneity: $Tau^2 = 0.01$; $Chi^2 = 38.49$, df = 10 ($P < 0.0001$); $I^2 = 74\%$

Test for overall effect: $Z = 1.30$ ($P = 0.19$)

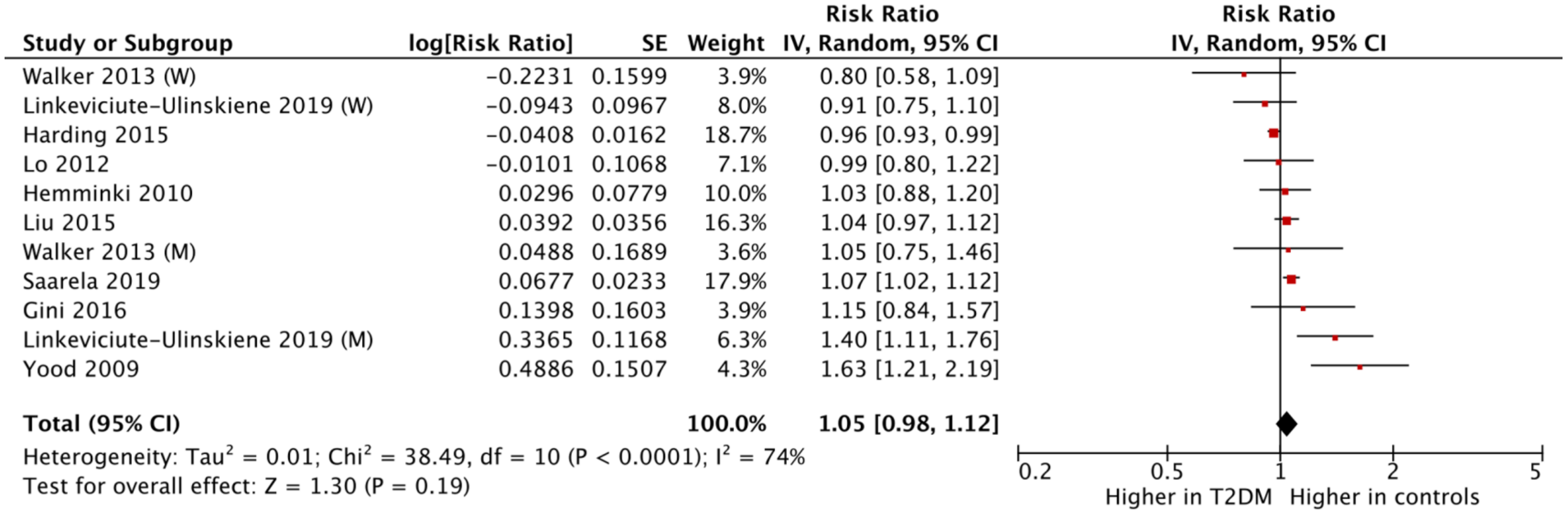

Figure S1. Forest plot of the association between type 2 diabetes and melanoma incidence. Only studies defining diabetes type as specifically type 2 diabetes are included in the analysis. Risk measurements including 95% Cis are reported on a logarithmic scale. IV: inverse variance. (M): Men. (W): Women.

Diabetes and melanoma in studies with scores of 8 or 9 on the Newcastle-Ottawa Scale

Study or Subgroup	log[Ratio]	SE	Weight	Ratio IV, Random, 95% CI
Tseng 2016	−0.7765	0.4806	0.6%	0.46 [0.18, 1.18]
Linkeviciute–Ulinskiene 2019 (W)	−0.0943	0.0967	9.4%	0.91 [0.75, 1.10]
Harding 2015	−0.0408	0.0162	21.4%	0.96 [0.93, 0.99]
Lo 2012	−0.0101	0.1068	8.3%	0.99 [0.80, 1.22]
Hemminki 2010	0.0296	0.0779	11.7%	1.03 [0.88, 1.20]
Saarela 2019	0.0677	0.0233	20.6%	1.07 [1.02, 1.12]
Atchison 2011	0.1222	0.0473	16.7%	1.13 [1.03, 1.24]
Attner 2012	0.1484	0.177	4.0%	1.16 [0.82, 1.64]
Linkeviciute–Ulinskiene 2019 (M)	0.3365	0.1168	7.4%	1.40 [1.11, 1.76]
**Total (95% CI)**			**100.0%**	**1.05 [0.97, 1.13]**

Heterogeneity: $Tau^2 = 0.01$; $Chi^2 = 33.89$, $df = 8$ ($P < 0.0001$); $I^2 = 76\%$

Test for overall effect: $Z = 1.16$ ($P = 0.24$)

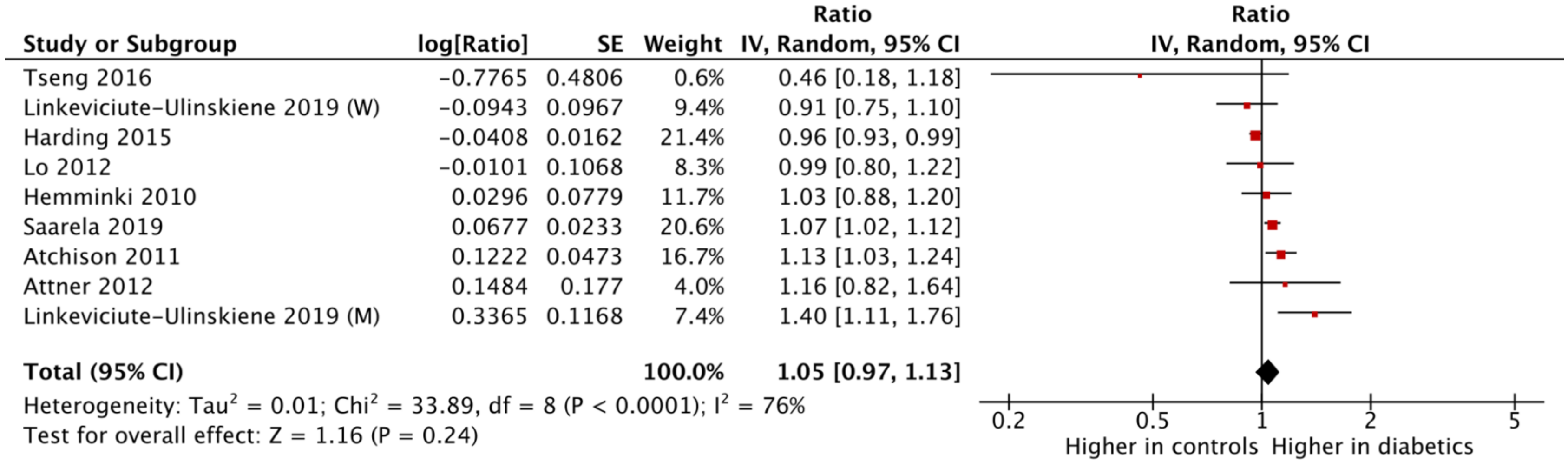

Figure S2. Forest plot of the association between diabetes and melanoma incidence. Only studies with a score of 8 or 9 on the Newcastle-Ottawa scale are included in the analysis. Risk measurements including 95% Cis are reported on a logarithmic scale. IV: inverse variance. (M): Men. (W): Women.

Diabetes and melanoma incidence in studies with more than 5 years of follow-up.

Study or Subgroup	log[Risk Ratio]	SE	Weight	Risk Ratio IV, Random, 95% CI
Linkeviciute–Ulinskiene 2019 (W)	−0.0943	0.0967	8.6%	0.91 [0.75, 1.10]
Harding 2015	−0.0408	0.0162	21.4%	0.96 [0.93, 0.99]
Wideroff 1997 (M)	0	0.182	3.4%	1.00 [0.70, 1.43]
Wideroff 1997 (W)	0	0.1139	7.0%	1.00 [0.80, 1.25]
Hemminki 2010	0.0296	0.0779	11.0%	1.03 [0.88, 1.20]
Saarela 2019	0.0677	0.0233	20.5%	1.07 [1.02, 1.12]
Atchison 2011	0.1222	0.0473	16.2%	1.13 [1.03, 1.24]
Wotton 2011 (1963–1998)	0.1398	0.2681	1.7%	1.15 [0.68, 1.95]
Attner 2012	0.1484	0.177	3.5%	1.16 [0.82, 1.64]
Linkeviciute–Ulinskiene 2019 (M)	0.3365	0.1168	6.7%	1.40 [1.11, 1.76]
**Total (95% CI)**			**100.0%**	**1.05 [0.98, 1.13]**

Heterogeneity: $Tau^2 = 0.01$; $Chi^2 = 31.45$, df = 9 (P = 0.0002); $I^2 = 71\%$
Test for overall effect: Z = 1.39 (P = 0.16)

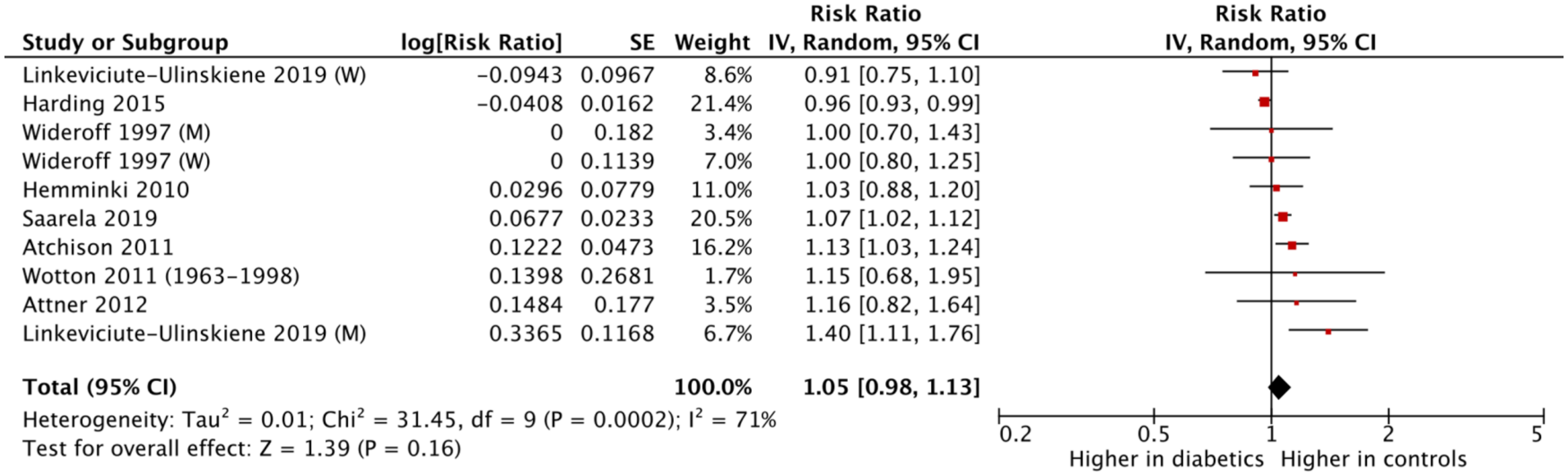

Figure S3. Forest plot of the association between diabetes and melanoma incidence. Only studies with a follow-up time of more than 5 years are included in the analysis. Risk measurements including 95% Cis are reported on a logarithmic scale. IV: inverse variance. (M): Men. (W): Women.
